# The Influence of Thawing Method on Bioactive Substances, Color, and Sensory Parameters in Frozen Strawberries from Retail Chains †

**DOI:** 10.3390/foods15193386

**Published:** 2026-09-23

**Authors:** Zuzana Megyesy Eftimová, Jana Zahumenská, Boris Semjon, Nikola Hudáková, Eva Dudriková

**Affiliations:** 1 Department of Food Hygiene, Technology and Safety, University of Veterinary Medicine and Pharmacy in Košice, Komenského 73, 041 81 Košice, Slovakia; zuzana.megyesy.eftimova@uvlf.sk (Z.M.E.); boris.semjon@uvlf.sk (B.S.); eva.dudrikova@uvlf.sk (E.D.); 2 Department of Morphological Disciplines, University of Veterinary Medicine and Pharmacy in Košice, Komenského 73, 041 81 Košice, Slovakia; nikola.hudakova@uvlf.sk

**Keywords:** strawberries, thawing, phenolic profile, antioxidant activity, sensory evaluation

## Abstract

Background: Freezing and thawing methods are critical to the final quality of food products. Methods: Our work focuses on evaluating the effect of five thawing protocols (in the refrigerator, at room temperature, in the microwave, and in a water bath at water temperatures of 20 and 40 °C) on the physicochemical properties, color, sensory properties, content of bioactive compounds, and antioxidant activity in frozen fruits of *Fragaria* spp. from different retail chains (*n* = 7) and from diverse countries of origin. Results: We found that although thawing at room temperature is a slow process, it best preserved the original quality of the strawberries when compared with other methods. Different thawing protocols had various effects on the color, sensory properties, polyphenol profile (TPC, TFC, TPA, and anthocyanins determined spectrophotometrically), and antioxidant activity (DPPH method) of strawberries. Our results indicate that each evaluated thawing method was viable. Conclusions: This study can provide a basis for selecting appropriate thawing processes in the strawberry processing industry based on the intended use of the food for further processing.

## 1. Introduction

The garden strawberry (*Fragaria* × *ananassa* L.), native to South America, is a hybrid species grown in open fields and greenhouses worldwide. It is one of the youngest and most widely consumed berries, valued not only for its taste and appearance but also for its numerous health benefits, recognized since ancient times [1]. Their high nutritional and dietary value, distinctive taste, and aroma are the qualities that have made strawberries popular among consumers. At the same time, these qualities make them a raw material suitable for direct consumption and for the production of frozen foods, juices, smoothies, jams, and compotes [2,3,4]. From a nutritional standpoint, strawberries contain a high proportion of easily soluble sugars, organic acids, folic acid, and micronutrients (vitamins A, B1, B2, and mineral salts, iron, calcium, phosphorus, potassium, zinc, magnesium, sulphur, boron, fluorine, cobalt, and molybdenum) [5,6]. Strawberries are also known for their high content of bioactive compounds (polyphenols, flavonoids, and anthocyanins) and high antioxidant activity, which give them high nutritional and commercial value [7]. Phenolic compounds in strawberries help determine overall quality form sensory properties and contribute to health benefits [8,9]. Strawberries exhibit a wide range of beneficial properties, such as hypolipidemic, antioxidant, anticancer, and anti-inflammatory effects [10,11]. Studies have confirmed that red berries, such as strawberries, have higher antioxidant activity than other fruits, including apples, peaches, pears, grapes, tomatoes, oranges, or kiwis [12]. Soil and climatic changes, fruit ripeness, variety, and post-harvest handling influence the content of bioactive compounds in strawberries [13,14]. Due to their high water content and delicate skin, strawberries are highly susceptible to mechanical damage and microbial infection (rot) after harvest, which leads to spoilage and consequently complicates storage and efficient transportation while significantly shortening shelf life [15].

A key factor affecting fruit availability in retail networks is seasonality; therefore, strawberries intended for processing are distributed in frozen form. High consumer demand for this food increases the need for large-scale production of frozen strawberries [16]. Frozen strawberries are the most common raw material used in industrial production [17]. Freezing, a common preservation technique, extends the shelf life of strawberries by inhibiting microbial growth and physicochemical reactions at low temperatures. Fruit quality in the frozen supply chain is constantly evolving [18]. However, freezing is significantly more effective at preserving the freshness and original characteristics of fruit than traditional methods such as drying or canning [19]. Freezing and subsequent thawing can affect fruit composition and, consequently, its quality (texture, taste, appearance, color, nutrients), which impacts its nutritional and sensory properties [16,20]. Since the thawing process is essential before consuming or further processing frozen strawberries, it is a key factor influencing the final product quality [21,22]. Freezing and thawing cause dehydration and subsequent rehydration of tissue; therefore, many similarities with the drying and rehydration of plant tissue can be expected [23,24,25]. Frozen foods are stored in storage facilities where temperatures are maintained at around −18 °C, the limit set by legislation (e.g., the European Directive on Deep-Frozen Foods) [26]. Defrosting is generally slower than freezing and is left to the consumer’s discretion. Consumers decide how to defrost food, whether using slow or fast methods. Traditional methods commonly used at home include thawing in a water bath, in the open air, or in the refrigerator [27]. However, consumers must be aware that the thawing process can lead to the reactivation of microbes and enzymes when the food is reheated [28]. Thawing at low temperatures better preserves product quality; however, it is inefficient, and the long duration prevents it from meeting practical production and consumption requirements [29,30]. Thawing food in a water bath is the fastest method, but it allows dissolved substances to leach into the surrounding liquid; this can be minimized by wrapping the food in plastic wrap [31]. Rapid thawing can also be achieved by electromagnetic means, such as microwave radiation, radiofrequency (RF) radiation, or resistive heating [32,33]. Microwave heating results in significantly uneven heating as the frozen part barely absorbs microwaves. The edges of the food may overheat, while the inner core remains frozen [32]. Selecting appropriate thawing technologies and optimal conditions is key to producing high-quality thawed strawberry products free from quality deterioration and microbial contamination [34]. Current literature provides a detailed overview of the quality parameters of strawberries and the mechanisms of their freezing and thawing, comprehensive knowledge regarding the impact of specific thawing methods on their color, sensory, physicochemical, and bioactive properties remains insufficiently elucidated. Previously published scientific studies have primarily focused on innovative and unconventional methods suitable for industrial processing. However, they have largely overlooked the standard procedures used by consumers at home. To address this knowledge gap, this study examines widely used thawing methods. It is important to note that each method of thermal thawing may have a different effect on the color, sensory properties, polyphenol content, and antioxidant activity of strawberries. The aim of this study was therefore to clarify and analyze the differences in strawberry quality through the comparison of associations between different thawing methods and post-thaw quality parameters. It provides a comprehensive analysis of the polyphenol content, antioxidant activity, color, and sensory profile in an effort to understand their stability during freezing and thawing and thus allow the prediction of strawberry quality acceptance.

## 2. Materials and Methods

### 2.1. Samples

The research included samples of frozen strawberries purchased from seven retail chains from different countries of origin and grown in the European Union. All samples are reported without mentioning the name of the producer. Immediately after purchase, the samples were transported to the Department of Food Hygiene, Technology, and Safety at the University of Veterinary Medicine and Pharmacy in Košice (UVMP), where they were stored in a freezer at −18 °C (ECM 22131W, Electrolux, Stockholm, Sweden). The samples were prepared before analysis using five thawing techniques.

### 2.2. Sample Preparation

A total of 105 sample analyses (7 retail chains × 5 thawing methods × 3 replicates) were conducted at the Department of Food Hygiene, Technology, and Safety at UVMP in Košice. The strawberry samples were purchased from retail stores in a deep-frozen state and stored at −18 °C in a freezer (model ECM 22131W, Electrolux, Stockholm, Sweden) until analysis. Before thawing, the strawberries were sorted, and those showing signs of mechanical damage or visible spoilage were discarded. The dimensions of the analyzed samples ranged from the smallest sample, measuring 19.52 mm in height and 17.34 mm in width, to the largest strawberry sample, measuring 54.23 mm in height and 40.15 mm in width. Only healthy berries of uniform size measuring 20–25 mm in height and 18–23 mm in width were used for the analysis. For the initial physicochemical characterization, the frozen strawberries were first thawed at room temperature for 2 h (24 °C), during which time the thawed berries reached 4 °C (±1 °C). The temperature was monitored in real time during thawing using a digital thermometer placed on a table near the thawing strawberries. Before analyzing the bioactive compounds, color, and sensory profile, the samples were prepared using five different thawing methods. The water-bath thawing method was applied twice: in the first case, the water temperature was 40 °C, and in the second case, 20 °C. The strawberries were placed in a polyethylene (PE) bag and submerged in a water container for 120 min. This step completely prevented direct contact between the fruit and the water, thereby avoiding undesirable leaching of bioactive compounds and mechanical damage to the fruit’s texture. After the elapsed time, the temperature of the strawberries was measured with a probe thermometer and found to have reached a constant temperature of 4 °C (±1 °C). Thawing was performed using a microwave oven (model MWD201/WH, Whirlpool, Hefei, China) at 400 W for 5 min. The frozen strawberries were evenly spread out in a single layer in the center of the rotating turntable inside the microwave cavity. Heating was paused after 2.5 min to measure the sample temperature with a probe thermometer. Thawing took place in a refrigerator (Model K337BAA, Gorenje, Kranj, Slovenia) for 24 h, maintaining a constant temperature of 4 °C and 87% humidity. The criterion for completing the thawing process was reaching a core temperature of 4 °C. After thawing, the strawberries were homogenized in a blender (ETA model 0010, Hlinsko, Czech Republic) and subsequently extracted in a 1:2 ratio (25 g strawberries; 50 mL solvent); the mixture was then shaken for 24 h on a shaker (LT3 Nedfrom s.r.o., Valašské Meziříčí, Czech Republic). The samples were then filtered. All chemicals used were of analytical grade and were purchased from Sigma-Aldrich (St. Louis, MO, USA) and CentralChem (Bratislava, Slovakia). Solutions for the determinations were prepared immediately before measurement. Each sample was extracted independently using 96% ethanol solution in triplicate, and the analyses were performed on the same day.

### 2.3. Physicochemical Analysis

Thawed strawberry samples were evaluated for sugar content, pH, and water activity (a_w_). Each physicochemical variable determination was performed in triplicate. Sugar content was measured using a digital refractometer (Atago PAL-87S, Atago USA, Bellevue, WA, USA) according to ISO 2173:2003 [35]. Before the measurement, the instrument was calibrated with distilled water by setting the value to 0 °Bx. The results were determined as average values.

The pH value was determined using a pH meter (pH-metrominoLab ^®^ 7110, Wissenschaftlich Technische Werkstätten, Weilheim, Germany) with a calibrated pH scale in pH units and a sample temperature of 20 °C according to ISO 1842:1991 [36]. Calibration was performed using commonly used buffers (pH 4.01, 7.01, and 10.01). Labmaster -a_w_ instrument (Novasina AG, Lechen, Switzerland) was used to measure water activity at a constant temperature of 25 °C according to the ISO 18787:2017 method [37].

The dry matter content was determined by drying the samples at 105 °C to constant weight in accordance with ISO 1442:2023 [38] (applied by analogy to plant material). The ash content was determined by incinerating the samples at 550 °C in accordance with ISO 936:1998 [39]. The content of nitrogenous substances (N-substances) was determined based on the nitrogen content using the Kjeldahl method (ISO 937:2023) [40], multiplied by a factor of 6.25. Crude fat content was determined by the extraction method specified in ISO 1443:1973 [41]. The determination of crude fiber, which quantifies the content of organic substances in feed insoluble in weakly acidic and weakly alkaline environments, was performed according to the method specified in ISO 6865:2000 [42]. The percentage of non-nitrogenous extractives (NFE) was determined by calculation as the residue of dry matter after subtracting the ash content, nitrogenous substances, crude fat, and crude fiber [43].

### 2.4. Total Polyphenol Content

Total polyphenol content (TPC) was determined by the Folin–Ciocalteu colorimetric method [44]. After the reaction of phenolic compounds with the Folin–Ciocalteu reagent, the absorbance was measured after 30 min in the dark against a blank sample with a spectrophotometer (T65 UV–Visible Spectrophotometer, PG Instruments Limited, Leicestershire, UK). The result was expressed as gallic acid equivalent (R^2^ = 0.9978) (mg·L^−1^ GAE) and calculated on a dry sample weight (mg GAE/100 g d.w.).

### 2.5. Total Flavonoid Content

Total flavonoid content (TFC) was determined by spectrophotometry according to Willett [45]. The absorbance of the samples was measured at the wavelength of 415 nm against a blank using a UV–vis spectrophotometer (T65 UV–Visible Spectrophotometer/UK). The flavonoid content was expressed based on a quercetin calibration curve (mg·L^−1^ QE) in mg QE/100 g dry weight (R^2^ = 0.9977).

### 2.6. Total Phenolic Acids Content

Total phenolic acid content (TPA) was determined spectrophotometrically according to Farmacopea Polska [46]. The absorbance of the samples was measured at a wavelength of 490 nm against a blank using a UV–vis spectrophotometer (T65 UV–Visible Spectrophotometer/UK). Caffeic acid was used as a standard (R^2^ = 0.999), and the results are expressed in mg·L^−1^ GAE and in mg·100 g^−1^ dry weight of caffeic acid equivalents per dry sample.

### 2.7. Total Anthocyanins Content

To determine the total anthocyanin content (TAC), a modified pH-difference method according to Lapornik, Prošek, and Wondra [47] was used. This method measures the increase in absorbance of the sample at reduced pH.

The volume of 1 mL of the extract was pipetted into two test tubes, and 1 mL of 0.01% HCl in 80% ethanol was added. Then, 10 mL of a 2% aqueous solution of HCl and 10 mL of a McIlvaine buffer solution with pH = 3.5 (c = 0.2 mol·dm^−3^ Na_2_HPO_4_ and c = 0.1 mol·dm^−3^ citric acid) were added. The absorbance of both solutions was measured at a wavelength of 520 nm. The total anthocyanin content was calculated from the difference in absorbance of both solutions, multiplied by the factor (*f* = 369.598) of the molecular weight of the standard anthocyanin targets and expressed in mg·L^−1^ of extract.

### 2.8. Antioxidant Activity

To measure antioxidant activity, a simple DPPH (2,2-diphenyl-1-picrylhydrazyl) radical uptake method was used. A 3.9 mL volume of DPPH solution was pipetted into the cuvette; the absorbance was then measured, corresponding to the initial concentration of the DPPH solution (A0). Subsequently, 0.1 mL of the sample was added, and after the reaction mixture had stood in the dark for 10 min, the absorbance was measured at a wavelength of 515 nm (At, where t = 10) using a spectrophotometer (T65 UV–Visible Spectrophotometer/UK). The efficiency of the sample was calculated according to the mathematical relationship: % inhibition = [(A_0_ − A_10_)/A_0_] × 100. Antioxidant activity was expressed as the % inhibition of DPPH radicals according to Brand-Williams [48].

### 2.9. Sensory Evaluation

Sensory evaluation of the experimental strawberry samples was performed at the University of Veterinary Medicine and Pharmacy in Košice (UVMP), Slovak Republic. The sensory panel consisted of 12 evaluators aged 26 to 50 years, selected from among the employees of the Department of Food Hygiene, Technology and Safety at UVMP. All evaluators had previous experience and training in descriptive food analysis. Before the evaluation itself, the panelists completed additional training focused on the sensory properties monitored in this study. The panelists evaluated the overall appearance, color, aroma, taste, texture, and overall acceptability using a 9-point hedonic scale ranging from 1—very unpleasant to 9—very pleasant [49]. During the training, individual descriptors of appearance, color, aroma, taste, texture, mouthfeel, and aftertaste were defined. The selection and definition of descriptors were carried out through discussion and consensus of the panel members. Subsequently, an evaluation sheet was created, containing all monitored sensory attributes. The panel was trained, and sensory analysis was performed using the principles of quantitative descriptive analysis (QDA) in accordance with the relevant sensory standards [50].

In the quantitative sensory analysis, the evaluators assessed the intensities of the following variables of color (pale yellow, intense red, dark red, and ruby red), aroma (distinct, pungent, odorless, strawberry, fruity, grassy, sweet, forest-like, honey-like, and undesirable odors), and taste (sweetness, acidity, fruitiness, freshness, juiciness, bitterness, astringency, and flavor intensity). Texture was described and analyzed in terms of firmness, juiciness (juicy), pithiness (resinous), softness, tenderness of the flesh, and the fibrous texture of the fruit. Aftertaste (intense, sweet, and sour) attributes were rated on a 10 cm intensity scale.

In the individual test, sensory evaluation was performed on individual halves of thawed fruit. In the individual test, panelists received 5 samples of fruit from 7 retail chains (Supplementary Figure S1). The samples were immediately placed in coded, sealed containers, and all samples were served at room temperature. The evaluators used filtered water to cleanse their palates between samples; each session allowed sufficient time for evaluation to minimize fatigue. The testing room was clean, well-lit, and odorless, and the temperature and relative humidity were controlled [51].

### 2.10. Colorimetric Determination of Color

Objective evaluation of the color of the experimental strawberry samples was carried out based on the color coordinates L*, a*, and b*. The measurement was carried out using a Chroma Meter CR-410 with a measuring surface with a diameter of 50 mm, D65 illumination, and a standard viewing angle of 2° (Konica Minolta Sensing Inc., Osaka, Japan) at room temperature. Before each measurement, the instrument was calibrated using the manufacturer-supplied calibration plate CR-A43. Subsequently, the measurement results of L*, a*, and b* of the CIELAB colorimetric system were recorded [52]. Six measurements were taken from each sample, and the values obtained were then statistically evaluated.

### 2.11. Statistical Analysis

The results obtained in this research were expressed as the mean and standard deviation (SD). Statistical data analysis was carried out via R-statistics software version 4.5.2 [53]. A two-way ANOVA was used with Tukey’s test for multiple comparisons of means at a significance level of *p* < 0.05. The effects of thawing (five methods of thawing strawberries) and solvent on the bioactive substances in strawberries were determined as the main factors of data analysis. Pearson’s correlation coefficient [54] was used to express the relationships between variables. Values ranging from −1 (negative correlation) to +1 (positive correlation) defined the relationships between the parameters. The coefficients were analyzed using GraphPad Prism 11 software (San Diego, CA, USA).

## 3. Results and Discussion

### 3.1. Results of Physicochemical Analyses of Various Strawberry Samples

The initial part of the research focused on identifying and evaluating the differences in chemical parameters of strawberry samples from different retail chains. The first part of the experimental work investigated the content of dry matter, nitrogenous substances, crude fat, crude fiber, ash, NFE (nitrogen-free extractable substances), sugar content, pH, and a_w_ in strawberries purchased from seven retail chains. Significant differences in these parameters can be caused by growing different cultivars, different management methods, and different agroclimatic conditions. The results showed statistically significant differences (*p* < 0.001) between individual samples for all monitored parameters, with the samples from retail chains C and D generally achieving higher values than the samples from other retail chains. The most significant differences were recorded for the content of dry matter, nitrogenous substances, crude fat, crude fiber, ash, and nitrogen-free extractable substances (NFE), for which the samples were clearly divided into different statistical groups (Table 1).

The chemical composition of the fruit refers to the content of all components, including water, and is important from both a nutritional and technological point of view. The chemical composition of the fruit is responsible for the formation of organoleptic, nutritional, and biological properties of the product [55]. High-quality varieties are considered to be those with the highest dry matter content, which indicates a higher nutrient level. The term “dry matter” includes all nutritional components of the fruit apart from water [56]. The nutritional composition of strawberries depends primarily on the variety, degree of maturity, fertility, and agrotechnical measures used [57].

Strawberries contain 92% water, sugars, minerals, vitamins, organic acids, and antioxidants, which are necessary for the normal functioning of the human body [58]. The authors reported that the chemical composition of strawberries is 8.3% carbohydrates, 0.8% proteins, 0.5% fat, 0.5% ash, and 89.9% water [59].

Karakashova et al. [56] reported that the highest total dry matter content was 14.43% in strawberries of the Mesecharka variety and the lowest value was 9.33% in strawberries of the Hummi Grande variety. In the Elsanta variety, the total dry matter content was 10.77%. Therefore, they concluded that the content of chemical substances in strawberries depends on the variety, degree of maturity and growing conditions. Similar findings were also reported by other authors [57].

Table 2 shows the results of the acidity determination of samples expressed as pH values of strawberries after thawing. During the observation period, pH values ranged from 3.44 ± 0.08 in the strawberry sample from retail chain C to 3.85 ± 0.08 in the strawberry sample from retail chain B. Strawberries are characterized by an acidic pH of 3.2 to 4.2. Zhou et al. [60] stated that acidic environments generally reduce growth, though specific exceptions can survive these conditions and alter local pH levels [61].

Water activity has a significant impact on the physical, mechanical, chemical, and microbiological properties and stability of food. If the a_w_ value is from 1.0 to 0.85, almost all microorganisms reproduce well because they have enough water and dissolved nutrients. Foods with a_w_ values below 0.70 are considered safe. Most often, these are dried foods and foods with high contents of fats, sugars, and salt. Nevertheless, it is always necessary to take into account that food spoilage or inactivation of microorganisms may occur [62]. The strawberry samples showed water activity values of 0.895 ± 0.00 to 0.905 ± 0.00 (*p* < 0.001), which represent a very suitable environment for microbial growth. García-Salinas et al. [63] monitored physicochemical parameters in strawberries during storage and concluded that significant fluctuations in the water activity level were observed throughout the storage until the 6th day, then stabilized until the 8th day, and then decreased until the 11th day. Therefore, the water activity of strawberries decreased during refrigerated storage. Water loss in the fruit is one of the main factors causing high susceptibility of strawberries to spoilage [64].

The sugar content in strawberries ranged from 6.70 ± 0.09 for the strawberry sample from retail chain C to 11.20 ± 0.18 for that from retail chain G. Reducing sugars (glucose and fructose) play an important role in the freshness and sweetness of the fruit. Therefore, determining them in fruit is important, as they indicate fruit ripeness. During fruit ripening, the glucose and fructose content increases due to the use of organic acids in gluconeogenesis, but this process leads to a decrease in the citric acid content in the fruit [65]. Consequently, the strawberry samples from retail chain C also showed signs of being unripe, they were yellow to green in color in places, which also reflected in the amount of sugar measured. In contrast, strawberries from retail chain G were a distinct dark red color and showed the highest sugar content. The research therefore showed that dark red strawberries had higher sugar concentrations, which are related to the qualitative characteristics of strawberries. In their study, Karakashova et al. [56] concluded that total sugar content varies depending on the variety, degree of maturity, and growing conditions.

Del Olmo [66] states in his study that the content of reducing sugars increases with storage time and that the sugar content determines the shelf life, freshness, and sweetness of strawberries. Simkova et al. [67] investigated the effects of freezing and subsequent thawing on the sugar content of strawberries. They concluded that glucose and fructose contents after thawing did not differ among the thawing treatments compared to frozen strawberries. However, the sucrose content differed significantly. Strawberries thawed in a microwave oven showed a higher sucrose content (33.9 mg/g), while the sucrose content of strawberries thawed at 4 °C for 24 h and at 20 °C for 6 h decreased by 66% and 37%, respectively, compared with that of frozen strawberries. Strawberries thawed at 37 °C for 2 h showed a sucrose content comparable to that of frozen strawberries (−20 °C for 4 weeks). The results showed no significant difference in sucrose content between freezing and storage.

Schwieterman et al. [68] suggest that freezing can cause changes in sugars, which can affect taste perception and change the taste of the fruit, including sweetness [69]. Castro et al. [70] and Moraga et al. [71] found that the sugar content decreased during storage in the freezer, which mainly affected the sucrose content. This phenomenon can be attributed to invertase activity, which hydrolyzes sucrose [72]. Skrede [73], in his study, found that the sucrose content in fruit decreased by 70% after thawing at 4 °C. Simkova et al. [67] reported similar results, stating that the highest sucrose losses in fruit were observed during thawing at 4 °C for 24 h, and they concluded that the loss of sucrose content depends on the thawing time more than on the thawing temperature.

In the present study, the sugar content was determined only in strawberries after using one method of thawing, namely, thawing at room temperature. Due to these differences, our findings are not directly comparable to those of the previously cited studies. Therefore, it can be concluded that there are significant differences between the sample groups of strawberries from different chains, with strawberries from chain C showing the lowest sugar content, pH, and a_w_, but the highest dry matter and nitrogen-free extract (NFE) content. These observations confirm the need to evaluate food composition and standardize criteria not only for food safety, but also for consumer satisfaction and awareness.

### 3.2. Results of Bioactive Compounds and Antioxidant Activity Analysis of Strawberry Samples (Influence of Retail Chain, Thawing, and Solvent)

Strawberries are known for their high content of phenols, anthocyanins, and ascorbic acid, which significantly contribute to their antioxidant activity [74]. The most abundant groups in strawberries are phenolic acids and anthocyanins, which are responsible for the color, flavor, and nutritional properties of strawberries [75]. Phenolic compounds are highly sensitive to light, heat, oxygen, and pH changes, leading to rapid degradation during processing [74]. The present study provides insights into variability in relationships among polyphenols, flavonoids, phenolic acids, anthocyanins, and antioxidant activity in frozen strawberries from seven retail chains thawed by five thawing methods using three solvents (Table 3).

In Table 3, the results of the determination of bioactive compounds and antioxidant activity in frozen strawberries purchased from retail chains are presented. Highly statistically significant differences at the *p* < 0.001 level were recorded among retail chains and thawing methods. The highest measured TPC value was in strawberries from retail chain E. The maximum TFC and TPA values were recorded in the sample group from retail chain B. The highest anthocyanin value was measured in samples from retail chain A, and the highest antioxidant activity was determined in strawberry samples from retail chain D.

It is difficult to compare the presented results with those of other authors because only a few authors address frozen strawberries purchased from retail chains, and, moreover, the retailers do not have detailed information on cultivation and varieties. The names of retail chains must remain anonymous, and because of this, it is unfeasible to compare the results with those of other authors, who studied either cultivars or growing locations.

Numerous previously published studies have examined the effects of variety and agroclimatic conditions on bioactive compounds in strawberries. These studies concluded that the content of bioactive compounds and the antioxidant capacity of strawberries can also be influenced by other parameters, such as harvest time, growing conditions, and environmental factors [76,77,78,79,80,81,82,83,84]. The results of chemical analysis (total polyphenol, flavonoid, phenolic acid, and anthocyanin content and antioxidant activity) of strawberry samples thawed in the refrigerator, at room temperature, in the microwave, and in a water bath at temperatures of 20 and 40 ° C are presented in Table 3.

The highest value of TFC was achieved by almost all samples thawed at room temperature except for the sample from retail chain D. As for the TFC content, it is not possible to say unequivocally which thawing protocol is the most suitable, but in three cases (retail chains C, G, and F) the highest measured values were obtained after the refrigerator-thawing method. Antioxidant activity showed a high percentage quenching rate in all monitored samples. For anthocyanins, it is also not possible to say unequivocally which thawing method is the most suitable, but the highest measured values were observed with thawing in a water bath at 40 °C (retail chains A, B, and G) and in a water bath at 20 °C (retail chains D and E). Significant differences were recorded between the total polyphenol content, antioxidant activity, and thawing method at the significance level of *p* < 0.001 (Table 3). The 20 °C water bath thawing method is the most suitable for retail chain D. However, based on the results, this thawing method is not unsuitable for bioactive substances.

Both an increase and a decrease in the content of phenolic compounds in strawberries during freezer storage have been found in several studies [17,85,86]. Although freezing did not have a strong negative effect on phenolic content, some thawing methods showed a significant loss of phenolic content. Holzwarth et al. [3] reported no significant effect on non-anthocyanin phenolic content during thawing. Simkova et al. [67] determined the total phenolic content in strawberry samples thawed in several ways. The fruits were thawed in a refrigerator at 4 °C for 24 h, at room temperature (24 °C) for 6 h, for 2 h in an electric oven at 37 °C, and in a microwave oven (600 W) for 5 min. The authors noted a significant decrease in total phenolic content after thawing in all cases, except microwave thawing, suggesting differences in enzymatic activity between cultivars. They found that among all thawing methods, fruits thawed at 24 °C showed a significant decrease in the content of all groups of phenolic compounds, which can be explained by the high activity of peroxidase (POD) and polyphenol oxidase (PPO) at this temperature. Li et al. [87] reported in their study that water-soluble bioactive compounds are potentially lost during thawing due to plasmolysis-induced drip loss.

Jiang et al. [30] found that slow thawing at room temperature aggravated enzymatic browning and oxidative damage, leading to significant quality loss. The best quality of strawberries was preserved by low-temperature thawing, but it required the longest thawing time and was the least efficient. In contrast, electric and magnetic field thawing achieved relatively rapid thawing while effectively preserving strawberry firmness, color, nutritional content, cell wall integrity, moisture distribution, and flavor properties, offering a favorable balance between thawing efficiency and quality preservation. Therefore, these two methods are recommended as the most suitable thawing technologies. Regarding microwave thawing, they found that the total phenolic content, anthocyanin content, and antioxidant activity of strawberries were most similar to those of fresh strawberries. This phenomenon may be due to microwave radiation disrupting plant cell structure, which promotes the release of bound phenolic compounds into an extractable, free state [30]. It may also account for the elevated antioxidant activity in the microwave-treated strawberry samples.

The correlation matrix (Figure 1) shows the Pearson correlation coefficients between the evaluated parameters and indicates the linear relationships between them. Strong positive correlations were found between TFC d.w. and TPA d.w. parameters (r = 0.68, *p* < 0.001) and between TPC d.w. and TPA d.w. (r = 0.53, *p* < 0.001). For TPC d.w. and TPA d.w., this is not surprising, since phenolic acids are a subset of phenolic compounds, and the correlation between TFC d.w. and TPA d.w. can be explained by common biosynthesis pathways [88]. Regarding antioxidant activity, we noted a strong positive relationship with another variable in only one case, namely with the amount of anthocyanins (r = 0.57, *p* < 0.001), which supports the claim that various anthocyanins are among the mediators of the antioxidant activity of strawberries [89].

### 3.3. Sensory Analysis of Different Strawberry Samples (Influence of Retail Chain and Thawing Protocol)

Sensory analysis showed differences between individual samples and thawing methods, especially in appearance, color, aroma, texture, juiciness, taste, and overall acceptability (Table 4, Figure 2). The appearance of the samples was generally evaluated positively, with no significant differences between individual samples, with the exception of a single sample from retail chain E. Strawberries from retail chains A, E, and G were very small in size, ripe to overripe, had a rather deep red to ruby color, were significantly grainy, and had soft flesh, resembling strawberries from the harvest in appearance. In contrast, strawberries from retail chains B, C, D, and F were large, red, unripe in places, poorer in texture after thawing, and very fibrous, but with a more pronounced taste. The most acceptable appearance was that of strawberries from retail chain A thawed in a water bath at 20 and 40 °C. It can be concluded that thawing in the refrigerator seemed, at first glance, to be the best method among the thawing methods used because there was no significant loss of juice; in some cases, the juice did not even leak from the strawberries (retail chains F, G, and A). During thawing at room temperature, a significant loss of juice was noticed. When heated in the microwave, the appearance of the strawberries remained dull in some cases, as if they had been cooked, and in others, they had not yet been sufficiently thawed. When evaluating the color after thawing, no differences were noticeable except for thawing in the microwave, after which the strawberries had a less saturated color and shine in all cases. In most cases, the strawberries were a beautiful red to ruby red; some were pale yellow, especially those from chain C, indicating unripe pieces. The aroma was more intense when thawing was performed in a microwave oven, while strawberries thawed at room temperature, in the refrigerator, and in a water bath seemed to be less aromatic. In terms of juiciness, there were significant differences between the strawberries, with strawberries from each retail chain being firmer and juicier, with soft flesh, except for strawberries from retail chain G. These strawberry samples were rather fibrous and soft. When evaluating the thawing method itself, the best acceptability among the studied methods was achieved by strawberries thawed using the microwave method. The best overall taste was observed in strawberries thawed in the refrigerator and in samples from retail chain A. It can be concluded that the best overall acceptability, compared with that of the other studied thawing methods, was achieved by strawberries thawed in a water bath at temperatures of 40 and 20 °C.

In terms of sensory profile, the evaluators often used adjectives such as dark red and ruby red. These descriptors suggest that the strawberries were mostly ripe, which is an important factor for consumers. The more intense and vivid the red color after thawing, the better, as it gives the impression that the fruit is ripe and makes the appearance more visually attractive. Regarding the perception of the smell, adjectives such as strawberry, fruity, and sweet (in retail chain E and the forest) were regularly used. “Strawberry” was one of the most frequently repeated descriptors of strawberries for smell. The smell was perceived as pleasant, with no undesirable odors. Regarding the description of the taste, the strawberries were described with adjectives such as sweet, sour, fruity, fresh, juicy, and intense. The recurring words juicy, fresh, and sweet in all of them point to a common perception of juiciness, freshness, and sweetness. Regarding the texture of strawberries, the most frequently used adjectives were juicy, soft, soft flesh, and fibrous, leading to an overall positive perception with a preference for softness and fleshiness. The taste of the samples evaluated was intense, sweet, and sour (Figure 3).

For consumers, the most important quality attributes are appearance (uniform bright red color, size, and shape), firmness, and flavor, with flavor being perceived through a combination of gustatory and olfactory (aromatic) sensations [90,91]. Sweetness, along with strawberry flavor and aroma, is the primary factors determining the overall acceptability and quality of strawberries. In our studies, as well as in the work of other authors, evaluators most frequently assessed these characteristics when determining the sensory profile [92,93,94]. Studies focusing on consumer perception emphasize that sensory quality (aroma, flavor, and texture) is a key factor influencing the acceptance of fresh fruit, including strawberries [95].

During the evaluation of relationships between parameters, the correlations between individual characteristics of thawed strawberries were also analyzed (Figure 4). There was no significantly strong positive/negative relationship between biochemical parameters (TPC, TPA, TAC) and sensory characteristics, nor between biochemical parameters and color. In addition to the expected strong negative correlation between sweet and sour aftertaste (r = −0.55, *p* < 0.001), a strong positive correlation was observed between fresh taste and flavor intensity (r = 0.59, *p* < 0.001), as well as between fresh taste and intense aftertaste (r = 0.56, *p* < 0.001).

### 3.4. Color of Different Strawberry Samples (Influence of Retail Chain and Thawing)

The color parameters L*, a*, and b*, representing the brightness, redness, and yellowness (surface and flesh) of strawberries from retail chains thawed by five different methods, are presented in Table 5. The changes in the color parameters L* (indicating lightness), a* (negative values correspond to green, while positive values correspond to red shades) and b* (negative values indicate blue shades, while positive values indicate yellow shades) were extracted from color images of the outer and inner surfaces of strawberry samples.

The colorimetric variables revealed significant differences between individual strawberry samples depending on the thawing method and the analyzed part (pulp, surface). The surface brightness values (L*) were significantly higher for samples from retail chains A, B, C, and D thawed using the water bath method. As for retail chains F and G, the values were higher for samples thawed under microwave conditions and for samples from retail chain E thawed in the refrigerator. The highest lightness values in these samples indicate a lighter surface coloration of the strawberries. Conversely, the lowest L* values were recorded for samples from retail chains A, D, and F when thawed by the refrigerator method, with these samples showing a darker appearance. The thawing method significantly affected luminance for some retail samples (samples from retail chains B, C, D, and F). The parameter a* (redness) was higher for the sample from retail chain F thawed in the refrigerator. The increased red intensity may be attributed to the higher content of anthocyanins, which are sensitive to processing and wrapping [96]. The thawing method did not have a significant effect on the a* parameter; however, a slight decrease in the values in a water bath at 40 °C was observed in three monitored samples from retail chains B, C, and D. In five of the seven samples thawed by the water bath method, the most noticeable changes were observed in the yellowness of the samples. Based on the monitored surface parameters of strawberries, the worst colorimetric variables were obtained for samples from retail chains B, C, and D, which indicates that the aforementioned strawberries were pale in color, which may also be due to their immaturity.

As for the pulp, the L* values were highest for samples from retail chains B, C, and D thawed using the water bath method, which was also the case for the surface of these samples. For sample G, the highest measured luminosity was obtained with the microwave thawing method, as was also observed for the surface. The parameter a* (red coloration) did not show significant differences in the pulp between the samples, indicating a relatively stable proportion of red pigment. In contrast, higher a* values were observed on the surface. The values b* (yellow color) were stable in most measurements, except for some parameters. Statistically significant differences were recorded between retail chains and thawing methods at the *p* < 0.001 level.

Regarding the green-to-red axis (a* parameter) across the surface and pulp tissues, strawberries from retail chains A and E did not show significant differences between samples. A different thawing method was suitable for each retail chain, and from the coloration perspective, it can be concluded that the water bath thawing method was not suitable in most cases because it led to the leaching of color pigments into the water.

Alterations in fruit quality attributes are strongly linked to storage temperature and relative humidity in strawberries during cold storage [97]. Color is a critical quality attribute of strawberries that could strongly affect retail chain preferences [98]. Vivid, highly saturated colors usually correspond to high nutritional value in the context of fruits and vegetables [99]. The red color of strawberries is mainly due to phenolic compounds, of which anthocyanins are responsible for the natural reddish pigment that is distributed throughout the fruit. Therefore, slight changes observed in fruit luminosity are more likely attributable to surface-level oxidative reactions affecting phenolic compounds than to direct enzymatic inactivation [100]. Previous studies have confirmed that the a* value of strawberries is highly correlated with anthocyanin content [101]. The thawing process intensifies the destruction and degradation of pigments (mainly anthocyanins). When the fruit is thawed in a water bath, pigments migrate into the water and leach out, along with juice loss from the sample [102]. Peng, Zhao, Wen, and Ni found that microwave thawing at 100 W, 200 W, and 300 W increased the L* value of mango [101,103]. The formation of micropores on and inside the strawberry tissue increases light scattering, thereby increasing the apparent brightness [104]. Jiang et al. [30] found in their study that all thawing methods significantly reduced the a* value of strawberries (*p* < 0.05). This phenomenon is primarily attributed to the destruction of the cellular structure during freeze–thaw cycles, leading to the degradation and loss of anthocyanins responsible for the characteristic red color of strawberries [105]. Jiang et al. [30] concluded that the thawing method significantly affected the physicochemical properties, moisture distribution, microstructure, and volatile metabolite composition of strawberries.

## 4. Conclusions

Current research primarily focuses on verifying the origin and variety of strawberries and examining the impact of various freezing methods on nutritional and sensory quality, while less attention is being paid to identifying suitable thawing methods. Selecting an appropriate thawing method is crucial for preserving the product’s nutritional quality. This study aimed to determine which of five selected thawing methods—refrigeration, room temperature, microwave, and water baths at 20 °C and 40 °C—would have the least impact on the qualitative and quantitative composition of the strawberries. The results indicated that the thawing methods significantly influenced physicochemical properties, bioactive compound content, sensory attributes, and color. We found that although thawing at room temperature is a slow process, it best preserved the strawberries’ original quality when compared with other methods. Water bath thawing at 40 °C and 20 °C proved most effective among the protocols at maintaining appearance, color, and texture, though these methods were less favorable regarding bioactive compound retention. In comparison to other methods, thawing at room temperature was the best method for preserving flavor, but it resulted in a significant change in texture due to increased water loss. Although microwave thawing yielded the best results regarding strawberry aroma and texture, the intense heat caused significant cellular damage, resulting in increased juice loss and color deterioration (a dull appearance and reduced red color intensity). In conclusion, regarding the preservation of nutritional quality, it is impossible to definitively identify the single most suitable thawing method for the consumer, as the choice depends on which product attributes the individual prioritizes. Future research will focus on a more detailed analysis of specific phenolic compounds—using reliable analytical techniques such as HPLC—and vitamin C levels, aiming to gain deeper insight into how thawing parameters affect quality retention and the health benefits for consumers.

## Figures and Tables

**Figure 1:**
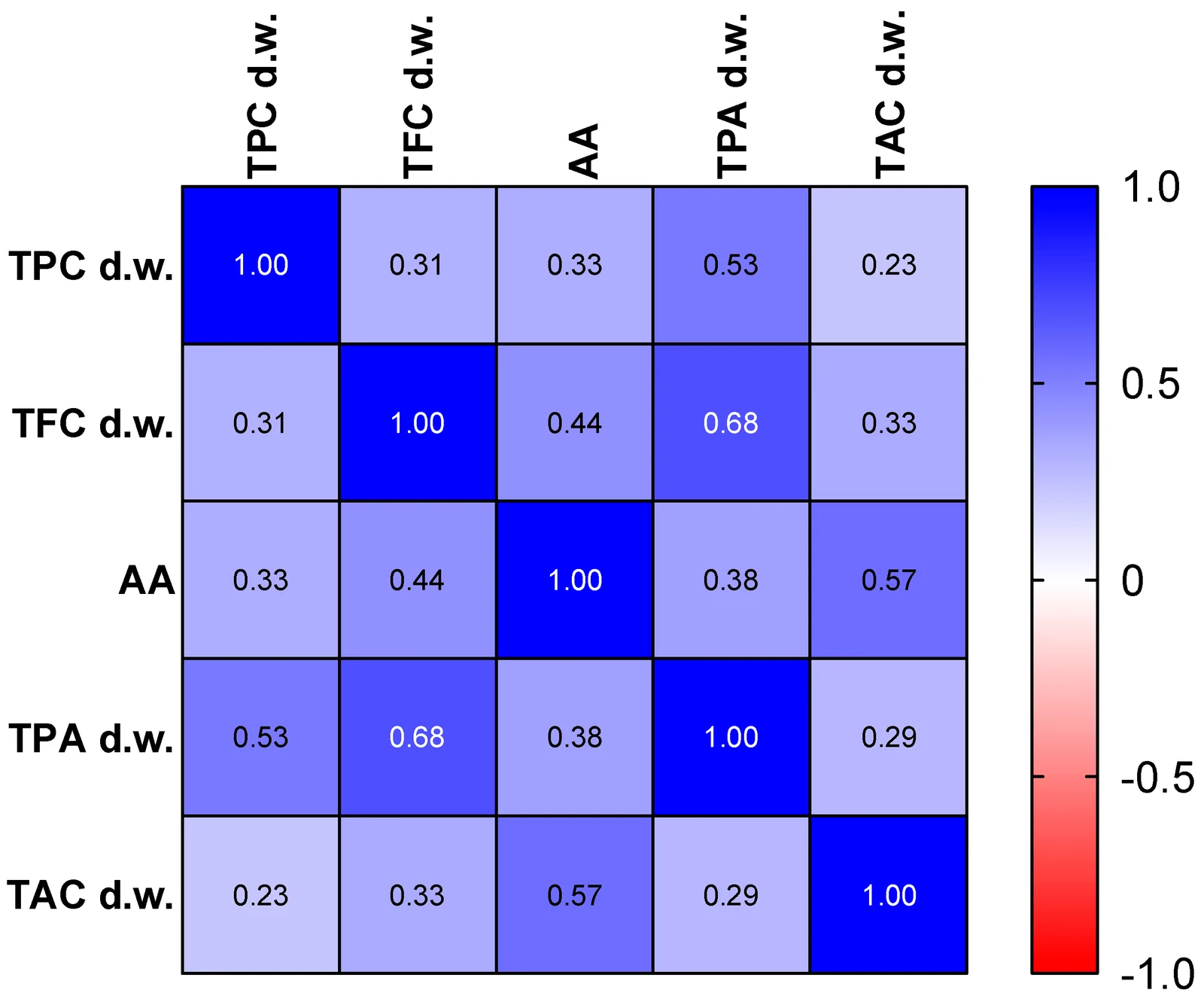
Correlation matrix of Pearson correlation coefficients between chemical parameters. The color gradient shows the magnitude of the Pearson correlation coefficient, with red indicating a negative correlation and blue indicating a positive correlation; the darker the color, the greater the absolute value of the coefficient. TPC: total polyphenol content; TFC: total flavonoid content; TPA: total phenolic acid content, TAC: total anthocyanin content; AA: antioxidant activity; d.w.: dry weight.

**Figure 2:**
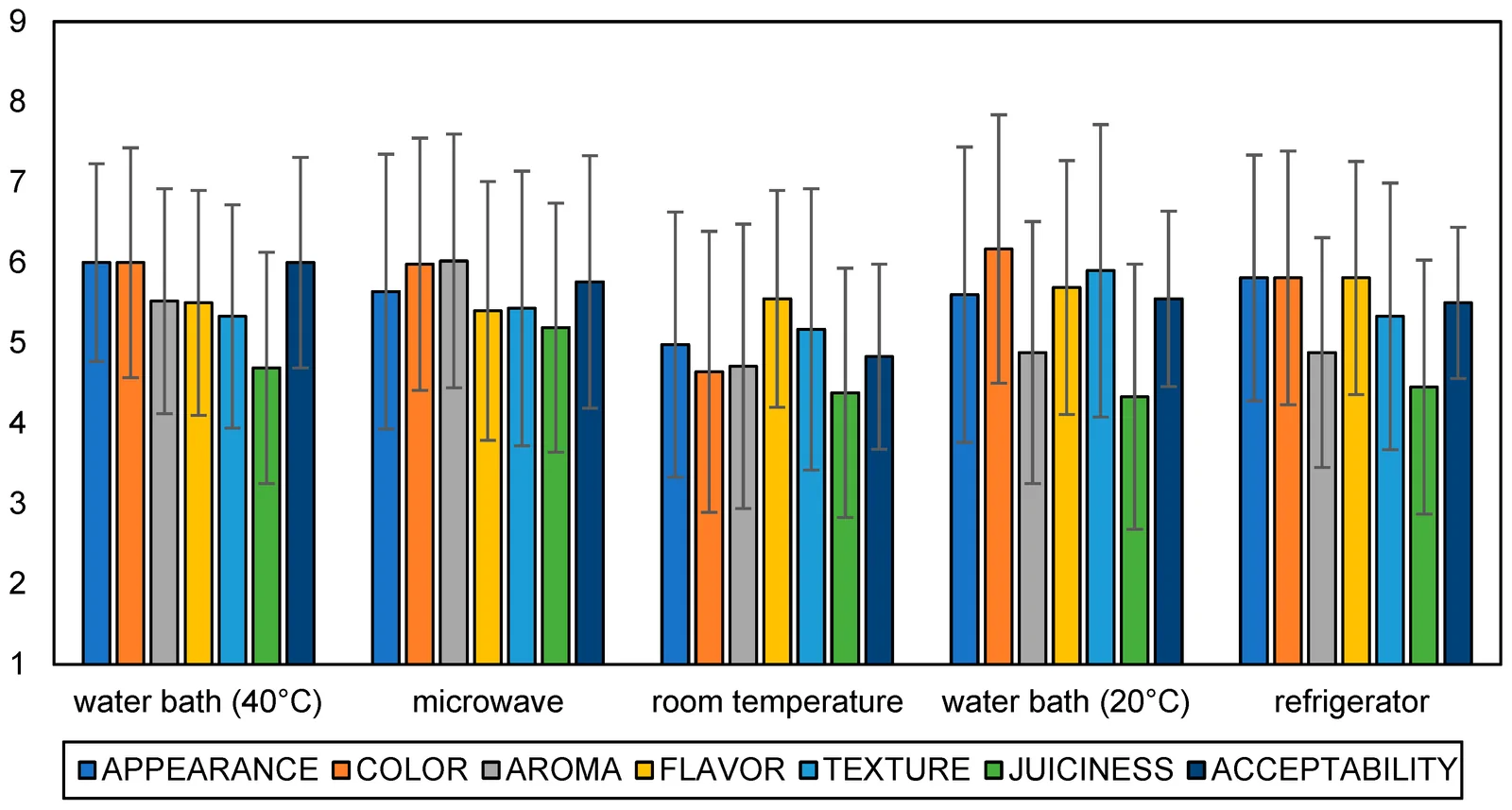
Results of sensory evaluation using a hedonic scale depending on the type of thawing (mean ± SD), showing the results of the hedonic evaluation of appearance, color, aroma, taste, texture, firmness, and overall acceptability of the strawberries evaluated.

**Figure 3:**
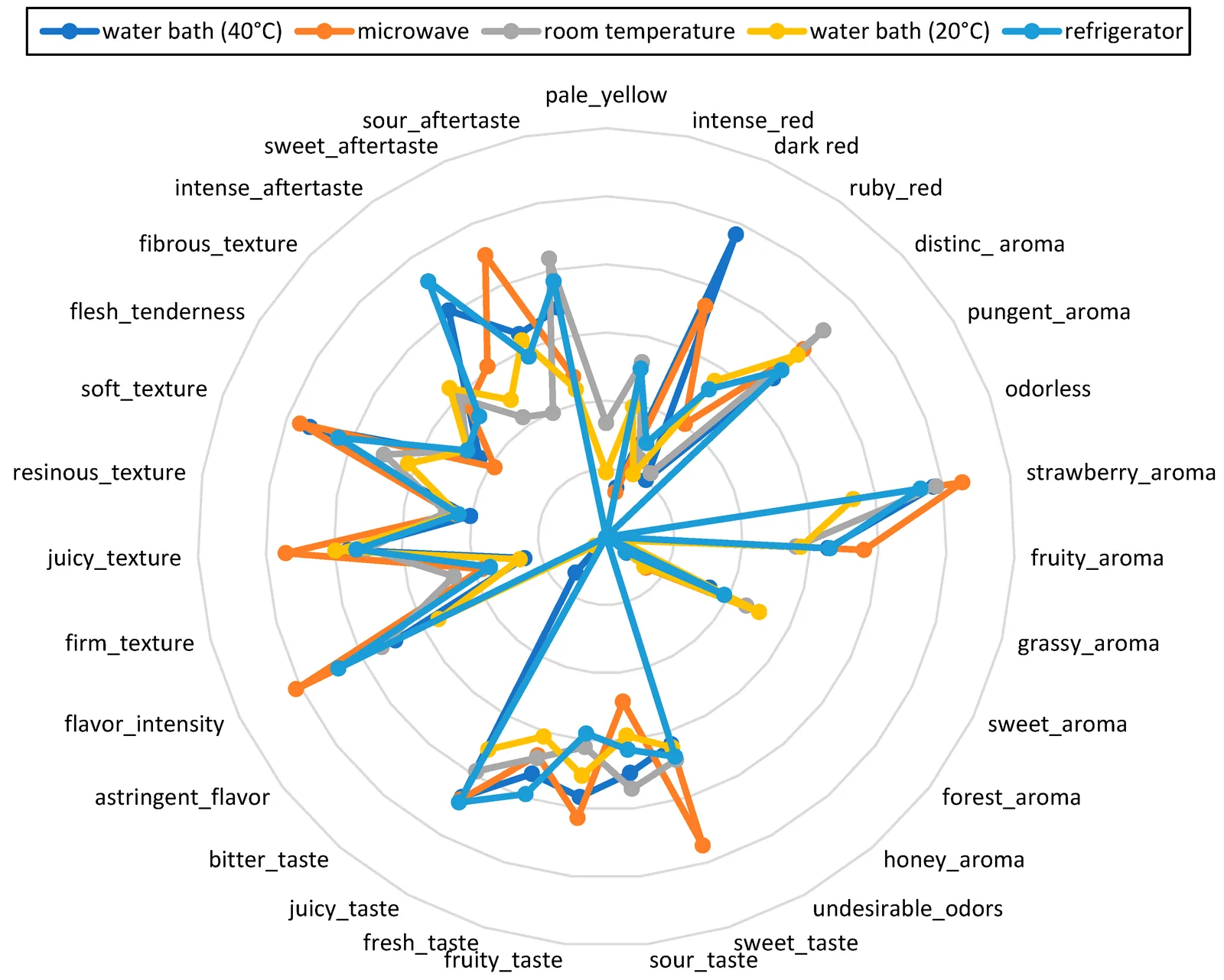
Sensory profile showing average intensity scores for descriptive strawberry properties from experimental strawberry samples thawed using 5 thawing methods.

**Figure 4:**
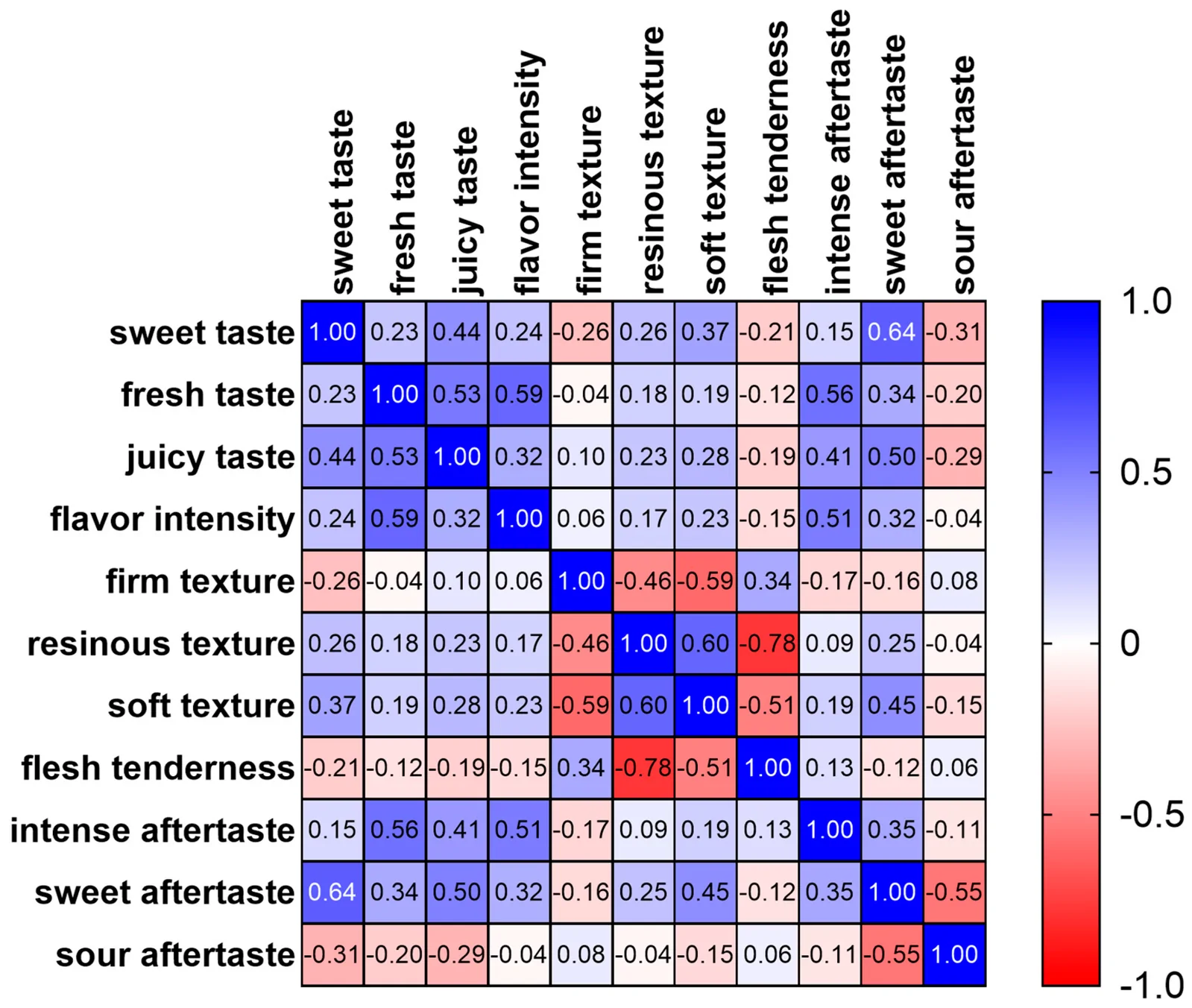
Pearson correlation coefficients between selected sensory parameters of thawed strawberries. The correlation matrix shows parameters with positive/negative correlations. The color gradient indicates the magnitude of the Pearson correlation coefficient (red—negative correlation; blue—positive correlation).

**Table 1:** Chemical parameters (dry matter, N-substances, crude fat, crude fiber, ash, and NFE) of thawed strawberries (% mean ± SD).

Retail Chain	Dry Matter	N-Substance	Crude Fat	Crude Fiber	Ash	NFE
A	102.44 ± 0.38 ^d^	6.81 ± 0.02 ^c^	2.40 ± 0.01 ^f^	10.92 ± 0.04 ^d^	4.60 ± 0.01 ^f^	77.49 ± 0.13 ^d^
B	104.81 ± 0.26 ^c^	6.21 ± 0.01 ^e^	2.50 ± 0.00 ^e^	11.50 ± 0.00 ^c^	5.80 ± 0.00 ^d^	78.82 ± 0.27 ^c^
C	144.23 ± 0.14 ^a^	8.79 ± 0.02 ^b^	3.60 ± 0.01 ^b^	11.68 ± 0.04 ^b^	10.03 ± 0.04 ^b^	110.21 ± 0.29 ^a^
D	140.31 ± 0.01 ^b^	9.90 ± 0.01 ^a^	4.91 ± 0.01 ^a^	12.92 ± 0.04 ^a^	10.80 ± 0.00 ^a^	101.67 ± 0.33 ^b^
E	84.44 ± 0.05 ^g^	5.42 ± 0.02 ^g^	2.91 ± 0.01 ^d^	8.63 ± 0.03 ^e^	3.91 ± 0.02 ^g^	63.63 ± 0.08 ^g^
F	93.51 ± 0.24 ^e^	5.51 ± 0.02 ^f^	3.40 ± 0.01 ^c^	8.10 ± 0.02 ^f^	8.31 ± 0.02 ^c^	68.11 ± 0.14 ^e^
G	84.83 ± 0.12 ^f^	6.49 ± 0.02 ^d^	2.41 ± 0.01 ^f^	6.62 ± 0.02 ^g^	5.31 ± 0.01 ^e^	64.22 ± 0.14 ^f^
	*p* < 0.001	*p* < 0.001	*p* < 0.001	*p* < 0.001	*p* < 0.001	*p* < 0.001

NFE: nitrogen-free extract; a–g: the means in columns with a different superscript letter are statistically different (*p* < 0.05).

**Table 2:** Chemical parameters (sugar, pH, law) of thawed strawberries (mean ± SD).

Retail Chain	pH	Sugar (°Brix)	a_w_
A	3.74 ± 0.02 ^bc^	8.30 ± 0.15 ^c^	0.901 ± 0.000 ^d^
B	3.85 ± 0.08 ^a^	7.10 ± 0.09 ^e^	0.905 ± 0.000 ^a^
C	3.44 ± 0.08 ^d^	6.70 ± 0.09 ^f^	0.895 ± 0.000 ^g^
D	3.69 ± 0.05 ^c^	7.27 ± 0.10 ^e^	0.902 ± 0.000 ^c^
E	3.72 ± 0.05 ^c^	7.60 ± 0.18 ^d^	0.903 ± 0.000 ^b^
F	3.83 ± 0.05 ^ab^	10.23 ± 0.19 ^b^	0.899 ± 0.000 ^e^
G	3.53 ± 0.04 ^d^	11.20 ± 0.18 ^a^	0.896 ± 0.000 ^f^
*p*-value	*p <* 0.001	*p* < 0.001	*p* < 0.001

a–g: the means in columns with a different superscript letter are statistically different (*p* < 0.05).

**Table 3:** Determination of bioactive substances and antioxidant activity regarding retail chain and thawing protocol (mean ± SD).

Retail Chain	TPC mg GAE·100 g^−1^ d.w.	TFC mg QE·100 g^−1^ d.w.	TPA mg CA·100 g^−1^ d.w.	TAC mg·100 g^−1^ d.w.	AA (% Inhibition DPPH)
Refrigerator					
A	1.60 ± 0.09 ^ijk^	0.26 ± 0.00 ^ijklmn^	2.66 ± 0.18 ^ijk^	441.61 ± 8.55 ^bc^	87.16 ± 0.33 ^def^
B	0.72 ± 0.04 ^p^	0.42 ± 0.02 ^b^	2.78 ± 0.13 ^ghij^	344.18 ± 13.80 ^fg^	90.45 ± 0.57 ^bc^
C	1.30 ± 0.05 ^lmno^	0.28 ± 0.01 ^hij^	2.28 ± 0.06 ^mn^	269.38 ± 6.96 ^klmn^	88.70 ± 0.56 ^cd^
D	1.14 ± 0.06 ^o^	0.35 ± 0.02 ^cd^	2.70 ± 0.12 ^hijk^	214.28 ± 12.97 ^pq^	88.91 ± 0.70 ^cd^
E	2.18 ± 0.12 ^cd^	0.34 ± 0.01 ^cd^	3.89 ± 0.18 ^a^	277.58 ± 15.98 ^jklm^	81.20 ± 0.99 ^k^
F	1.26 ± 0.06 ^mno^	0.29 ± 0.01 ^fgh^	3.81 ± 0.15 ^a^	243.17 ± 16.81 ^nop^	84.76 ± 0.47 ^ghi^
G	0.89 ± 0.01 ^p^	0.33 ± 0.01 ^de^	3.26 ± 0.09 ^bcd^	307.61 ± 18.69 ^hij^	76.17 ± 1.70 ^n^
Room temperature					
A	2.19 ± 0.14 ^cd^	0.32 ± 0.01 ^de^	3.12 ± 0.14 ^bcdef^	452.00 ± 17.07 ^b^	85.98 ± 0.30 ^efg^
B	2.31 ± 0.16 ^bc^	0.36 ± 0.01 ^c^	2.94 ± 0.15 ^efghi^	250.59 ± 17.38 ^mno^	71.24 ± 0.95 ^pq^
C	1.33 ± 0.04 ^lmno^	0.23 ± 0.01 ^opq^	2.09 ± 0.05 ^n^	293.14 ± 8.92 ^ijkl^	91.56 ± 0.99 ^ab^
D	1.90 ± 0.08 ^efgh^	0.23 ± 0.01 ^opq^	3.26 ± 0.13 ^bcd^	261.60 ± 7.67 ^lmno^	69.83 ± 1.39 ^q^
E	2.23 ± 0.08 ^bcd^	0.27 ± 0.01 ^hijkl^	3.38 ± 0.20 ^b^	414.98 ± 29.91 ^cd^	81.73 ± 0.99 ^jk^
F	2.31 ± 0.09 ^bc^	0.22 ± 0.00 ^q^	3.12 ± 0.12 ^bcdef^	337.87 ± 5.58 ^fgh^	83.38 ± 1.19 ^ij^
G	2.06 ± 0.12 ^de^	0.24 ± 0.01 ^mnopq^	2.88 ± 0.19 ^efghi^	264.27 ± 19.73 ^klmn^	84.95 ± 0.91 ^ghi^
Microwave					
A	1.81 ± 0.10 ^fgh^	0.32 ± 0.01 ^de^	2.98 ± 0.14 ^defgh^	501.29 ± 19.13 ^a^	90.07 ± 0.41 ^bc^
B	1.93 ± 0.07 ^efg^	0.31 ± 0.01 ^efg^	3.29 ± 0.12 ^bc^	114.74 ± 4.93 ^st^	76.52 ± 1.34 ^mn^
C	1.32 ± 0.06 ^lmno^	0.26 ± 0.02 ^ijklmn^	2.14 ± 0.16 ^n^	237.00 ± 11.72 ^nopq^	72.29 ± 1.22 ^op^
D	1.45 ± 0.08 ^klm^	0.22 ± 0.01 ^q^	2.59 ± 0.07 ^jkl^	165.22 ± 8.32 ^r^	76.86 ± 1.10 ^mn^
E	1.84 ± 0.07 ^fgh^	0.29 ± 0.01 ^fghi^	3.10 ± 0.02 ^bcdef^	410.50 ± 23.62 ^cd^	83.48 ± 0.48 ^hij^
F	2.15 ± 0.11 ^cd^	0.24 ± 0.01 ^mnopq^	2.84 ± 0.04 ^fghij^	310.00 ± 19.61 ^hij^	84.95 ± 0.83 ^ghi^
G	1.86 ± 0.08 ^efgh^	0.26 ± 0.01 ^jklmno^	2.46 ± 0.14 ^klm^	265.59 ± 13.84 ^klmn^	84.77 ± 0.93 ^ghi^
Water bath 20 °C					
A	1.33 ± 0.04 ^lmno^	0.23 ± 0.01 ^pq^	1.32 ± 0.04 ^o^	357.60 ± 10.21 ^ef^	91.46 ± 0.39 ^ab^
B	1.49 ± 0.10 ^jkl^	0.61 ± 0.02 ^a^	3.88 ± 0.18 ^a^	389.15 ± 21.18 ^de^	87.78 ± 0.58 ^de^
C	1.18 ± 0.08 ^no^	0.23 ± 0.02 ^opq^	1.20 ± 0.02 ^o^	229.63 ± 14.34 ^opq^	78.04 ± 1.10 ^lmn^
D	2.07 ± 0.10 ^de^	0.40 ± 0.02 ^b^	3.37 ± 0.04 ^b^	292.87 ± 10.82 ^ijkl^	93.13 ± 0.50 ^a^
E	2.02 ± 0.10 ^def^	0.27 ± 0.01 ^hijk^	3.35 ± 0.18 ^b^	459.18 ± 12.16 ^b^	59.32 ± 2.12 ^r^
F	1.82 ± 0.07 ^fgh^	0.24 ± 0.01 ^mnopq^	3.12 ± 0.18 ^bcdef^	86.67 ± 4.37 ^t^	70.07 ± 1.09 ^q^
G	1.70 ± 0.08 ^hij^	0.26 ± 0.02 ^ijklm^	2.37 ± 0.09 ^lmn^	314.23 ± 7.00 ^ghi^	73.66 ± 1.22 ^o^
Water bath 40 °C					
A	1.31 ± 0.08 ^lmno^	0.25 ± 0.02 ^klmnop^	2.47 ± 0.05 ^klm^	529.84 ± 29.94 ^a^	79.60 ± 0.16 ^kl^
B	2.40 ± 0.17 ^b^	0.35 ± 0.01 ^cd^	3.94 ± 0.09 ^a^	430.13 ± 4.79 ^bc^	71.11 ± 1.29 ^pq^
C	1.46 ± 0.10 ^klm^	0.23 ± 0.00 ^pq^	2.09 ± 0.07 ^n^	208.53 ± 8.43 ^q^	58.98 ± 0.31 ^r^
D	1.88 ± 0.05 ^efgh^	0.23 ± 0.01 ^nopq^	2.87 ± 0.10 ^efghij^	212.76 ± 7.41 ^pq^	70.16 ± 1.03 ^pq^
E	2.67 ± 0.19 ^a^	0.31 ± 0.01 ^ef^	3.89 ± 0.12 ^a^	122.25 ± 7.99 ^s^	85.60 ± 0.40 ^fgh^
F	1.80 ± 0.06 ^ghi^	0.24 ± 0.01 ^lmnopq^	3.14 ± 0.19 ^bcde^	295.34 ± 11.16 ^ijk^	78.44 ± 0.64 ^lm^
G	1.38 ± 0.05 ^lmn^	0.28 ± 0.01 ^ghij^	3.06 ± 0.15 ^cdefg^	337.12 ± 15.52 ^fgh^	86.23 ± 0.64 ^efg^
*p*-value: S	*p* < 0.001	*p* < 0.001	*p* < 0.001	*p* < 0.001	*p* < 0.001
*p*-value: T	*p* < 0.001	*p* < 0.001	*p* < 0.001	*p* < 0.001	*p* < 0.001
*p*-value: S × T	*p* < 0.001	*p* < 0.001	*p* < 0.001	*p* < 0.001	*p* < 0.001

S: sample factor; T: thawing method factor; TPC: total polyphenol content; TFC: total flavonoid content; TPA: total phenolic acid content; TAC: total anthocyanin content; AA: antioxidant activity; d.w.: dry weight. GAE: gallic acid equivalent; QE: quercetin equivalent; CA: caffeic acid. a–t: means in columns with different superscript letters are statistically different (*p* < 0.05).

**Table 4:** The results of sensory evaluation using the hedonic scale depending on retail chain and thawing method (mean ± SD).

Samples	Appearance	Color	Aroma	Flavor	Texture	Fruitness	Overall Acceptability
Refrigerator							
A	6.67 ± 1.63 ^ab^	6.50 ± 1.76 ^abc^	6.00 ± 1.41 ^ab^	6.33 ± 1.03 ^ab^	5.50 ± 0.84 ^abcde^	5.50 ± 1.87 ^abc^	6.00 ± 0.63 ^abcd^
B	6.33 ± 1.51 ^ab^	6.17 ± 1.47 ^abcd^	4.83 ± 0.75 ^ab^	6.00 ± 1.41 ^ab^	5.33 ± 1.75 ^abcde^	5.67 ± 0.82 ^abc^	5.67 ± 0.52 ^abcde^
C	5.33 ± 1.03 ^ab^	5.50 ± 1.05 ^abcde^	4.17 ± 0.75 ^ab^	5.67 ± 1.37 ^ab^	4.50 ± 1.05 ^cde^	4.17 ± 0.75 ^abcd^	5.67 ± 0.52 ^abcde^
D	5.33 ± 1.03 ^ab^	5.50 ± 1.05 ^abcde^	4.17 ± 0.75 ^ab^	5.67 ± 1.37 ^ab^	4.50 ± 1.05 ^cde^	4.17 ± 0.75 ^abcd^	5.67 ± 0.52 ^abcde^
E	4.67 ± 1.03 ^ab^	4.50 ± 0.84 ^bcde^	5.17 ± 1.72 ^ab^	4.67 ± 1.51 ^ab^	5.17 ± 1.17 ^abcde^	3.50 ± 1.38 ^bcd^	5.00 ± 1.41 ^abcde^
F	5.83 ± 1.17 ^ab^	5.00 ± 2.00 ^abcde^	4.67 ± 2.16 ^ab^	5.83 ± 2.14 ^ab^	4.17 ± 1.33 ^de^	5.67 ± 1.37 ^abc^	5.33 ± 1.37 ^abcde^
G	6.50 ± 2.43 ^ab^	7.50 ± 1.05 ^a^	5.17 ± 1.60 ^ab^	6.50 ± 1.05 ^ab^	8.17 ± 0.75 ^a^	2.50 ± 0.84 ^d^	5.17 ± 1.17 ^abcde^
Room temperature							
A	5.33 ± 0.82 ^ab^	5.33 ± 1.37 ^abcde^	6.33 ± 1.63 ^ab^	5.67 ± 1.03 ^ab^	6.17 ± 2.14 ^abcde^	4.33 ± 1.97 ^abcd^	4.83 ± 0.75 ^bcde^
B	5.50 ± 2.43 ^ab^	4.67 ± 1.51 ^abcde^	5.00 ± 1.79 ^ab^	5.33 ± 2.07 ^ab^	3.50 ± 0.84 ^e^	3.17 ± 0.75 ^cd^	4.17 ± 0.75 ^de^
C	4.17 ± 1.17 ^ab^	3.00 ± 0.63 ^e^	3.83 ± 1.47 ^b^	4.83 ± 0.75 ^ab^	4.83 ± 1.17 ^bcde^	4.50 ± 1.38 a^bcd^	4.67 ± 1.03 ^cde^
D	4.17 ± 1.17 ^ab^	3.00 ± 0.63 ^e^	3.83 ± 1.47 ^b^	4.83 ± 0.75 ^ab^	4.83 ± 1.17 b^cde^	4.50 ± 1.38 ^abcd^	4.67 ± 1.03 ^cde^
E	4.17 ± 0.75 ^ab^	4.00 ± 1.10 ^cde^	3.83 ± 0.98 ^b^	5.67 ± 0.82 ^ab^	4.67 ± 1.03 ^cde^	3.33 ± 1.21 ^cd^	4.17 ± 1.17 ^de^
F	5.50 ± 2.17 ^ab^	5.83 ± 2.04 ^abcde^	4.67 ± 1.37 ^ab^	5.67 ± 1.37 ^ab^	4.67 ± 1.75 ^cde^	5.50 ± 1.05 ^abc^	5.67 ± 0.82 ^abcde^
G	6.00 ± 1.79 ^ab^	6.67 ± 0.82 ^abc^	5.50 ± 2.43 ^ab^	6.83 ± 1.60 ^ab^	7.50 ± 1.05 ^abc^	5.33 ± 1.75 ^abcd^	5.67 ± 1.63 ^abcde^
Microwave							
A	6.00 ± 1.41 ^ab^	7.00 ± 0.89 ^ab^	7.33 ± 1.51 ^a^	6.17 ± 1.47 ^ab^	5.83 ± 2.23 ^abcde^	6.83 ± 1.47 ^a^	7.17 ± 1.33 ^ab^
B	6.83 ± 1.72 ^ab^	6.83 ± 1.17 ^abc^	5.67 ± 1.51 ^ab^	6.17 ± 2.14 ^ab^	7.83 ± 0.98 ^ab^	4.50 ± 0.55 ^abcd^	6.00 ± 1.67 ^abcd^
C	5.83 ± 1.17 ^ab^	6.33 ± 0.82 ^abc^	5.67 ± 1.37 ^ab^	5.33 ± 1.03 ^ab^	5.33 ± 1.51 ^abcde^	5.17 ± 1.60 ^abcd^	5.83 ± 1.17 ^abcde^
D	5.83 ± 1.17 ^ab^	6.33 ± 0.82 ^abc^	5.67 ± 1.37 ^ab^	5.33 ± 1.03 ^ab^	5.33 ± 1.51 ^abcde^	5.17 ± 1.60 ^abcd^	5.83 ± 1.17 ^abcde^
E	3.50 ± 0.55 ^b^	3.33 ± 0.82 ^de^	5.00 ± 1.55 ^ab^	4.67 ± 0.82 ^ab^	4.17 ± 0.75 ^de^	4.17 ± 1.47 ^abcd^	3.50 ± 1.38 ^e^
F	5.50 ± 2.07 ^ab^	5.50 ± 1.87 ^abcde^	5.50 ± 1.05 ^ab^	4.17 ± 1.83 ^b^	4.33 ± 1.37 ^de^	567 ± 1.97 ^abc^	5.33 ± 1.03 ^abcde^
G	6.00 ± 2.00 ^ab^	6.50 ± 1.05 ^abc^	7.33 ± 1.63 ^a^	6.00 ± 2.00 ^ab^	5.17 ± 0.75 ^abcde^	4.83 ± 0.75 ^abcd^	6.67 ± 0.52 ^abc^
Water bath 20 °C							
A	7.17 ± 1.17 ^a^	6.83 ± 0.75 ^abc^	4.17 ± 1.47 ^ab^	7.33 ± 1.63 ^a^	5.83 ± 1.47 ^abcde^	5.17 ± 1.47 ^abcd^	6.17 ± 0.98 ^abcd^
B	6.17 ± 2.04 ^ab^	7.33 ± 1.37 ^ab^	6.17 ± 1.83 ^ab^	5.33 ± 1.03 ^ab^	5.83 ± 1.60 ^abcde^	5.33 ± 1.97 ^abcd^	6.50 ± 1.05 ^abcd^
C	5.00 ± 1.79 ^ab^	5.17 ± 1.72 ^abcde^	3.83 ± 1.17 ^b^	5.00 ± 1.41 ^ab^	5.83 ± 1.72 ^abcde^	3.50 ± 0.55 ^bcd^	4.83 ± 0.75 ^bcde^
D	5.00 ± 1.79 ^ab^	5.17 ± 1.72 ^abcde^	3.83 ± 1.17 ^b^	5.00 ± 1.41 ^ab^	5.83 ± 1.72 ^abcde^	3.50 ± 0.55 ^bcd^	4.83 ± 0.75 ^bcde^
E	4.33 ± 0.52 ^ab^	6.33 ± 0.52 ^abc^	5.83 ± 1.83 ^ab^	5.00 ± 1.41 ^ab^	5.50 ± 1.52 ^abcde^	4.33 ± 1.51 ^abcd^	5.33 ± 1.03 ^abcde^
F	5.00 ± 1.41 ^ab^	4.83 ± 2.04 ^abcde^	5.17 ± 0.98 ^ab^	5.67 ± 1.97 ^ab^	4.33 ± 2.07 ^de^	6.00 ± 1.26 ^abc^	6.00 ± 0.89 ^abcd^
G	6.50 ± 2.43 ^ab^	7.50 ± 1.05 ^a^	5.17 ± 1.60 ^ab^	6.50 ± 1.05 ^ab^	8.17 ± 0.75 ^a^	2.50 ± 0.84 ^d^	5.17 ± 1.17 ^abcde^
Water bath 40 °C							
A	7.00 ± 1.41 ^a^	6.33 ± 1.51 ^abc^	6.83 ± 0.75 ^ab^	7.00 ± 0.63 ^ab^	6.17 ± 0.75 ^abcde^	6.33 ± 1.37 ^ab^	7.33 ± 1.03 ^a^
B	5.83 ± 1.17 ^ab^	6.33 ± 0.52 ^abc^	6.50 ± 1.38 ^ab^	4.17 ± 0.98 ^b^	5.00 ± 1.90 ^bcde^	4.33 ± 1.51 ^abcd^	5.33 ± 1.37 ^abcde^
C	6.50 ± 1.05 ^ab^	6.67 ± 1.21 ^abc^	5.33 ± 0.82 ^ab^	5.67 ± 0.82 ^ab^	5.50 ± 1.05 ^abcde^	4.67 ± 1.03 ^abcd^	6.50 ± 1.05 ^abcd^
D	6.50 ± 1.05 ^ab^	6.67 ± 1.21 ^abc^	5.33 ± 0.82 ^ab^	5.67 ± 0.82 ^ab^	5.50 ± 1.05 ^abcde^	4.67 ± 1.03 ^abcd^	6.50 ± 1.05 ^abcd^
E	5.17 ± 0.75 ^ab^	4.00 ± 1.10 ^cde^	4.17 ± 1.17 ^ab^	5.00 ± 1.26 ^ab^	3.83 ± 0.75 ^de^	3.50 ± 0.55 ^bcd^	5.00 ± 0.63 ^abcde^
F	5.17 ± 0.75 ^ab^	5.17 ± 0.98 ^abcde^	4.83 ± 1.72 ^ab^	5.17 ± 1.60 ^ab^	4.50 ± 1.05 ^cde^	5.83 ± 1.17 ^abc^	5.50 ± 1.64 ^abcde^
G	5.83 ± 1.47 ^ab^	6.83 ± 1.17 ^abc^	5.67 ± 1.37 ^ab^	5.83 ± 1.94 ^ab^	6.83 ± 0.75 ^abcd^	3.50 ± 0.84 ^bcd^	5.83 ± 0.98 ^abcde^
*p*-value: S	*p* < 0.001	*p* < 0.001	*p* < 0.001	*p* < 0.001	*p* < 0.001	*p* < 0.001	*p* < 0.001
*p*-value: T	*p* < 0.001	*p* < 0.001	*p* < 0.001	*p* < 0.001	*p* < 0.001	*p* < 0.001	*p* < 0.001
*p*-value: S × T	*p* < 0.001	*p* < 0.001	*p* < 0.001	*p* < 0.001	*p* < 0.001	*p* < 0.001	*p* < 0.001

S: samples factor; T: thawing method factor. Different letters (a–e) indicate statistically significant differences (*p* < 0.05).

**Table 5:** Evaluation of color parameters of thawed strawberries (mean ± SD).

Retail Chain	L* Pulp	a* Pulp	b* Pulp	L* Surface	a* Surface	b* Surface
Refrigerator						
A	39.64 ± 0.97 ^lmnopq^	34.51 ± 1.07 ^defgh^	19.61 ± 0.23 ^klmn^	32.62 ± 0.47 ^klmno^	31.00 ± 0.32 ^hijk^	13.53 ± 0.56 ^ijkl^
B	39.45 ± 0.18 ^mnopq^	33.31 ± 0.12 ^ghij^	20.15 ± 0.17 ^ijk^	33.16 ± 0.32 ^ijklm^	29.53 ± 0.24 ^klm^	13.19 ± 0.19 ^jkl^
C	42.93 ± 0.17 ^efgh^	37.95 ± 0.35 ^ab^	23.37 ± 0.30 ^a^	33.12 ± 0.48 ^ijklm^	32.30 ± 0.38 ^efgh^	14.53 ± 0.62 ^hij^
D	38.21 ± 0.41 ^q^	34.10 ± 0.10 ^efghi^	19.12 ± 0.01 ^lmnop^	32.32 ± 0.13 ^lmnop^	33.64 ± 0.29 ^bcde^	14.69 ± 0.16 ^hi^
E	43.18 ± 0.10 ^efg^	33.12 ± 0.12 ^ghijk^	20.66 ± 0.34 ^ghij^	34.62 ± 0.50 ^hij^	32.21 ± 0.24 ^efghi^	14.96 ± 0.05 ^ghi^
F	39.34 ± 0.04 ^nopq^	39.13 ± 0.12 ^a^	22.94 ± 0.04 ^ab^	36.69 ± 0.41 ^fg^	30.53 ± 0.56 ^ijkl^	16.27 ± 0.36 ^fg^
G	42.21 ± 0.16 ^fghij^	35.63 ± 0.07 ^cde^	21.82 ± 0.07 ^cdef^	34.60 ± 0.01 ^hij^	34.35 ± 0.09 ^bc^	15.59 ± 0.07 ^gh^
Room temperature						
A	39.92 ± 0.36 ^lmnop^	36.39 ± 1.12 ^bc^	21.31 ± 0.36 ^efg^	28.63 ± 0.22 ^r^	27.34 ± 0.58 ^no^	7.76 ± 0.70 ^n^
B	41.65 ± 0.24 ^ghijk^	35.94 ± 0.31 ^cde^	22.57 ± 0.15 ^abcd^	32.85 ± 0.61 ^jklm^	33.90 ± 0.21 ^bcde^	14.39 ± 0.33 ^hij^
C	39.92 ± 0.36 ^mnop^	36.39 ± 1.12 ^bc^	21.31 ± 0.36 ^efg^	28.63 ± 0.22 ^r^	27.34 ± 0.58 ^no^	7.76 ± 0.70 ^n^
D	42.57 ± 1.05 ^efghi^	35.31 ± 0.68 ^cdef^	21.95 ± 0.56 ^cdef^	30.77 ± 1.15 ^nopq^	32.53 ± 1.07 ^defgh^	12.32 ± 1.34 ^l^
E	41.27 ± 0.19 ^hijkl^	33.64 ± 0.11 ^fghi^	21.16 ± 0.10 ^efgh^	35.23 ± 0.02 ^gh^	37.41 ± 0.03 ^a^	19.13 ± 0.01 ^cd^
F	39.93 ± 0.01 ^lmnop^	34.44 ± 0.23 ^defgh^	18.74 ± 0.09 ^nop^	40.85 ± 0.60 ^b^	31.02 ± 0.09 ^hijk^	20.36 ± 0.21 ^bc^
G	47.43 ± 0.38 ^d^	32.39 ± 1.33 ^ijkl^	20.00 ± 1.46 ^jkl^	38.75 ± 0.97 ^cde^	32.44 ± 0.40 ^defgh^	14.83 ± 0.13 ^ghi^
Microwave						
A	41.65 ± 0.24 ^ghijk^	35.94 ± 0.31 ^cde^	22.57 ± 0.15 ^abcd^	32.85 ± 0.61 ^jklm^	33.90 ± 0.21 ^bcde^	14.39 ± 0.33 ^hij^
B	42.70 ± 1.45 ^efghi^	29.61 ± 1.46 ^no^	20.32 ± 0.21 ^hijk^	34.35 ± 0.83 ^hijk^	28.00 ± 0.30 ^mn^	14.89 ± 1.00 ^ghi^
C	46.13 ± 2.35 ^d^	29.69 ± 1.98 ^no^	21.20 ± 0.47 ^efgh^	33.69 ± 1.44 ^hijkl^	23.30 ± 0.83 ^q^	14.39 ± 1.10 ^hij^
D	44.17 ± 1.16 ^e^	32.96 ± 0.59 ^hijkl^	22.60 ± 0.81 ^abc^	33.06 ± 0.40 ^ijklm^	29.10 ± 0.83 ^lm^	13.81 ± 1.05 ^ijk^
E	40.47 ± 0.13 ^klmno^	36.02 ± 0.23 ^cd^	21.65 ± 0.17 ^def^	33.05 ± 0.11 ^ijklm^	34.14 ± 0.16 ^bcd^	14.68 ± 0.09 ^hi^
F	49.67 ± 0.45 ^c^	27.86 ± 0.45 ^op^	19.44 ± 0.30 ^klmno^	32.68 ± 0.97 ^jklmn^	32.89 ± 1.78 ^cdef^	14.50 ± 0.27 ^hij^
G	47.59 ± 0.33 ^d^	27.63 ± 0.09 ^p^	16.28 ± 0.05 ^r^	40.70 ± 0.27 ^bc^	31.56 ± 0.33 ^fghij^	17.30 ± 0.19 ^ef^
Water bath 20 °C						
A	41.05 ± 0.63 ^ijklm^	38.34 ± 0.95 ^a^	22.06 ± 0.42 ^bcde^	29.76 ± 0.43 ^qr^	31.15 ± 0.41 ^ghijk^	10.60 ± 0.05 ^m^
B	38.98 ± 0.17 ^opq^	32.33 ± 0.19 ^ijkl^	17.05 ± 0.11 ^qr^	31.47 ± 0.48 ^mnopq^	25.59 ± 0.19 ^p^	10.46 ± 0.03 ^m^
C	50.83 ± 0.46 ^c^	30.06 ± 0.35 ^mn^	19.06 ± 0.25 ^mnop^	37.66 ± 0.96 ^ef^	35.31 ± 0.40 ^b^	18.58 ± 0.43 ^de^
D	39.44 ± 0.56 ^mnopq^	34.71 ± 0.50 ^cdefgh^	20.59 ± 0.82 ^ghij^	38.35 ± 0.16 ^def^	30.02 ± 0.25 ^jkl^	14.39 ± 0.10 ^hij^
E	43.00 ± 1.08 ^efg^	34.70 ± 0.57 ^cdefgh^	21.07 ± 0.43 ^fghi^	30.34 ± 0.36 ^qr^	35.31 ± 0.88 ^b^	14.51 ± 0.46 ^hij^
F	43.77 ± 0.57 ^ef^	31.42 ± 0.72 ^klmn^	17.29 ± 0.17 ^q^	34.87 ± 1.68 ^ghi^	32.85 ± 0.02 ^cdefg^	14.76 ± 0.52 ^hi^
G	39.81 ± 0.60 ^lmnopq^	31.61 ± 0.25 ^jklm^	18.89 ± 0.06 ^nop^	33.19 ± 2.12 ^ijklm^	25.82 ± 1.34 ^op^	10.77 ± 0.31 ^m^
Water bath 40 °C						
A	42.57 ± 1.05 ^efghi^	35.31 ± 0.68 ^cdef^	21.95 ± 0.56 ^cdef^	30.77 ± 1.15 ^nopq^	32.53 ± 1.07 ^defgh^	12.32 ± 1.34 ^l^
B	57.33 ± 1.19 ^b^	21.94 ± 2.08 ^q^	20.64 ± 0.40 ^ghij^	44.35 ± 2.32 ^a^	26.25 ± 1.95 ^op^	24.47 ± 1.80 ^a^
C	58.43 ± 1.11 ^b^	19.62 ± 0.98 ^r^	19.84 ± 0.52 ^jklm^	38.09 ± 0.13 ^def^	29.02 ± 1.20 ^lmn^	20.00 ± 0.78 ^cd^
D	61.40 ± 1.11 ^a^	17.08 ± 1.56 ^s^	15.32 ± 0.32 ^s^	40.90 ± 0.18 ^b^	32.62 ± 1.45 ^defgh^	21.75 ± 0.45 ^b^
E	38.45 ± 0.06 ^pq^	34.93 ± 0.26 ^cdefg^	18.52 ± 0.17 ^op^	30.55 ± 0.13 ^pqr^	34.94 ± 0.52 ^b^	13.53 ± 0.30 ^ijkl^
F	42.52 ± 0.12 ^efghij^	31.18 ± 0.18 ^lmn^	18.40 ± 0.15 ^p^	39.63 ± 0.88 ^bcd^	30.23 ± 0.58 ^jkl^	14.49 ± 0.46 ^hij^
G	40.85 ± 0.23 ^jklmn^	30.07 ± 0.02 ^mn^	18.54 ± 0.10 ^op^	30.70 ± 1.38 ^opq^	29.10 ± 0.58 ^lm^	12.41 ± 0.30 ^kl^
*p*-value: S	*p* < 0.001	*p* < 0.001	*p* < 0.001	*p* < 0.001	*p* < 0.001	*p* < 0.001
*p*-value: T	*p* < 0.001	*p* < 0.001	*p* < 0.001	*p* < 0.001	*p* < 0.001	*p* < 0.001
*p*-value: S × T	*p* < 0.001	*p* < 0.001	*p* < 0.001	*p* < 0.001	*p* < 0.001	*p* < 0.001

S: sample factor; T: thawing method factor; L*: brightness; a*: redness; b*: yellowness. Values are presented as the mean ± standard deviation. Different superscript letters (a–s) within a column indicate statistically significant differences (*p* < 0.05).

## Data Availability

The original contributions presented in this study are included in the article and Supplementary Materials. Further inquiries can be directed at the corresponding author.

## Supplementary Materials

The following supporting information can be downloaded at https://www.mdpi.com/article/10.3390/foods15193386/s1. Figure S1: Sensory key for evaluating the sensory properties of thawed strawberries. Letters A–G indicate samples from retail chains; numbers express thawing methods, namely water bath 40 °C (1), room temperature (2), samples thawed using a microwave (3), water bath 20 °C (4), and refrigerator thawing (5).
