# Supplementary material for: The Influence of Thawing Method on Bioactive Substances, Color, and Sensory Parameters in Frozen Strawberries from Retail Chains
Source: Foods. 2026 Sep 23;15(19):3386. doi: 10.3390/foods15193386 (PMC13664137; doi:10.3390/foods15193386)
Supplement: Supplementary file 1 [file foods-15-03386-s001.zip › foods-4542910-supplementary.pdf]

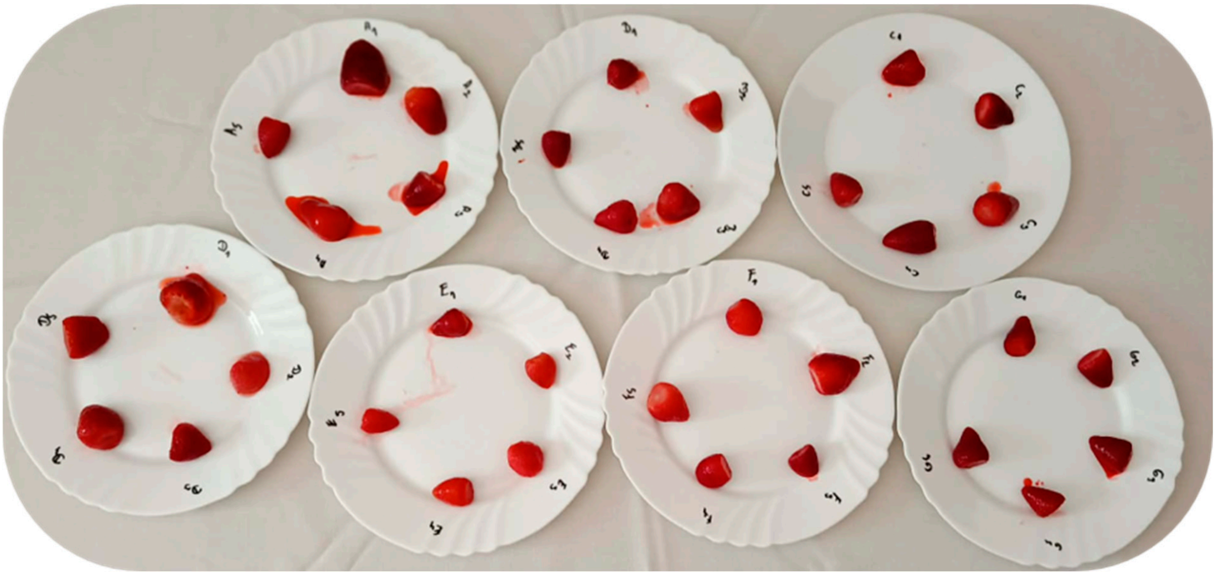

Supplementary Figure S1. Sensory key for evaluating the sensory properties of thawed strawberries. Letters A-G indicate samples from retail chains, numbers express thawing methods, namely water bath 40 °C (1), room temperature (2), samples thawed using a microwave (3), water bath 20 °C (4), and refrigerator thawing (5).
